# Characterization of Cardiovascular Alterations Induced by Different Chronic Cisplatin Treatments

**DOI:** 10.3389/fphar.2017.00196

**Published:** 2017-05-08

**Authors:** Esperanza Herradón, Cristina González, José A. Uranga, Raquel Abalo, Ma I. Martín, Visitacion López-Miranda

**Affiliations:** ^1^Área de Histología Humana y Anatomía Patológica, Departamento de Ciencias Básicas de la Salud, Universidad Rey Juan CarlosAlcorcón, Spain; ^2^Unidad Asociada ICDCi del Instituto de Química Médica, Consejo Superior de Investigaciones CientíficasMadrid, Spain; ^3^Grupo Interdisciplinar de Investigación en Dolor iCDol, Universidad Rey Juan Carlos-Banco de SantanderAlcorcón, Spain; ^4^Área de Histología Humana y Anatomía Patológica, Departamento de Ciencias Básicas de la Salud, Universidad Rey Juan CarlosAlcorcón, Spain

**Keywords:** cisplatin, cardiac toxicity, vascular toxicity, endothelial dysfunction, autonomic neuropathy

## Abstract

In the last years, many clinical studies have revealed that some cisplatin-treated cancer survivors have a significantly increased risk of cardiovascular events, being cisplatin-induced cardiovascular toxicity an increasing concern. The aim of the present work was to evaluate the cardiovascular alterations induced by different chronic cisplatin treatments, and to identify some of the mechanisms involved. Direct blood pressure, basal cardiac (left ventricle and coronary arteries) and vascular (aortic and mesenteric) functions were evaluated in chronic (5 weeks) saline- or cisplatin-treated male Wistar rats. Three different doses of cisplatin were tested (1, 2, and 3 mg/kg/week). Alterations in cardiac and vascular tissues were also investigated by immunohistochemistry, Western Blot, and or quantitative RT-PCR analysis. Cisplatin treatment provoked a significant modification of arterial blood pressure, heart rate, and basal cardiac function at the maximum dose tested. However, vascular endothelial dysfunction occurred at lower doses. The expression of collagen fibers and conexin-43 were increased in cardiac tissue in cisplatin-treated rats with doses of 2 and 3 mg/kg/week. The expression of endothelial nitric oxide synthase was also modified in cardiac and vascular tissues after cisplatin treatment. In conclusion, chronic cisplatin treatment provokes cardiac and vascular toxicity in a dose-dependent manner. Besides, vascular endothelial dysfunction occurs at lower doses than cardiac and systemic cardiovascular toxicity. Moreover, some structural changes in cardiac and vascular tissues are also patent even before any systemic cardiovascular alterations.

## Introduction

Cancer is among the leading causes of death worldwide. However, due to advances in early recognition and novel treatment modalities, cancer survival is improving. Unfortunately, with the improvement in morbidity and mortality rates due to cancer, the increase in long-term cardiac and vascular toxicity associated with cancer treatments has become an important concern. Cardiovascular side effects negatively impact quality of life and survival of patients. Moreover, the development of toxicity may require adjustments or discontinuation of the chemotherapy regimen, leading to worse outcomes. As such, early recognition of cardiovascular dysfunctions associated to the different chemotherapeutic agents becomes imperative (Curigliano et al., 2012; Hamo and Bloom, 2015; Denegri et al., 2016).

*Cis*-dichlorodiamine platinum (cisplatin) is an effective and widely used chemotherapeutic agent against various solid tumors, including those affecting the bladder, testicles, and ovaries (Mollman, 1990). Sensory neuropathy and renal damage are two of the common side effects that limit therapy with cisplatin. Neural damage provoked by cisplatin involves central, peripheral, and autonomic nerves (Boogerd et al., 1990). In relation to renal injury, it has been described that this antitumoral agent causes tubular damage and tubular dysfunction with sodium, potassium, and magnesium wasting, provoking, depending on the dose administered, a reversible or an irreversible decrease in glomerular filtration (Yao et al., 2007).

Cardiovascular alterations, such as hypertension, hyperlipidemia, and coronary artery disease or myocardial infarction are additional less frequent, but equally important, factors that have been also associated with platinum-based chemotherapy. These events do not appear to be dose dependent and they may occur during treatment, shortly after treatment, or in some cases months or years after completion of chemotherapy (Pai and Nahata, 2000).

In the past few years, cisplatin-induced vascular toxicity has become an increasing concern, affecting up to 12% of patients (Gospodarowicz, 2008; Ishioka et al., 2008; Vaughn et al., 2008). Given the efficacy of cisplatin, vascular toxicity represents a significant survivorship issue. Both endothelial dysfunction of large arteries and vascular neuropathy have been discussed as etiological factors for cisplatin-induced vascular alterations (Turlapaty and Altura, 1980; Rosenfeld and Broder, 1984; Samuels et al., 1987; Serrano-Castro et al., 2000; Pretnar-Oblak et al., 2007).

Animal models exist for cisplatin-induced sensory peripheral neuropathy (Verdú et al., 1999; Authier et al., 2003; Vera et al., 2007), or cisplatin induced-nephrotoxicity (Vickers et al., 2004) and there are experimental data about the cardiac toxicity produced by acute administration of this antitumoral agent (Al-Majed et al., 2006; Yüce et al., 2007). However, to our knowledge, no experimental data has been reported so far about the cardiovascular alterations that may be developed after chronic administration of cisplatin.

The aim of the present experimental work was to evaluate the possible cardiovascular alterations induced by different chronic cisplatin treatments (at doses at which it causes neuropathy, Vera et al., 2007). Modifications on blood pressure and heart rate (HR), basal heart function, such as conduit and resistance vascular reactivity were investigated. Besides, structural and molecular mechanisms involved in these alterations were also studied.

## Materials and Methods

The Ethical Committee at Universidad Rey Juan Carlos (URJC) approved the study. Experimental procedures were carried out in accordance with the recommendations of this Committee as well as with the EU directive for the protection of animals used for scientific purpose (2010/63/UE) and Spanish regulations (RD 109 53/2013).

### Animals

Male Wistar rats [240–300 g, Harlan-Iberica (Barcelona, Spain)] were placed in cages (4–6 animals) and maintained in environmentally controlled conditions (temperature of 20°C; humidity of 60%) with a 12 h light/12 h dark cycle. Animals were allowed free access to standard laboratory rat chow (Harlan-Iberica, Barcelona, Spain) and tap water, which was refreshed every day.

### Treatments

After a week of adaptation to the controlled conditions, the animals were divided into four treatment groups (10–15 animals per group), saline (0.9% NaCl) and cisplatin (1, 2, and 3 mg/kg, cumulative dose of 5, 10, and 15 mg/kg, respectively). Saline or cisplatin were administered intraperitoneally once a week for five experimental weeks. The maximum injection volume administered intraperitoneally in the animals was 0.5 ml. Before each antineoplastic drug injection, 2 ml of sterile saline solution was given subcutaneously to prevent renal damage via hyperhydration (Authier et al., 2003).

The doses of cisplatin were chosen based on those commonly used in experimental protocols in rats to induce a wide range of toxic effects caused by this anticancer agent that are also observed in humans (Authier et al., 2003; Malik et al., 2006; Vera et al., 2007; Cabezos et al., 2010).

### Blood Pressure and Heart Rate Measurements in Anesthetized Rats

In all the animals of the different experimental groups, after anesthesia with sodium pentobarbital (50 mg/kg intraperitoneally), a catheter coupled to a pressure transducer was inserted into the right carotid artery of the animals for direct measurements of systolic (SBP) and diastolic arterial blood pressure (DBP) and HR using a PowerLab/4e system (PanLab S.L., Barcelona, Spain). Recording of these cardiovascular parameters lasted for 10 min (Abalo et al., 2011).

After blood pressure measurements, the animals were euthanized, and the following preparations and experiments were performed.

### Isolated Heart Preparation

The hearts were removed and immersed into ice-cold modified Krebs-Henseleit buffer with the following composition: 118 mM NaCl, 4.7 mM KCl, 1.2 mM MgSO_4_, 1.2 mM KH_2_PO_4_, 2.5 mM CaCl_2_, 25 mM NaHCO_3_, and 10 mM glucose, and 2 nM pyruvate.

Afterward, they were immediately mounted on a Langendorff set-up, the aortic stump was cannulated and the heart was retrogradely perfused with the modified Krebs-Henseleit buffer. The buffer was kept at 37°C in water-heated jacketed chambers and gassed with 95% O_2_/5% CO_2_. The perfusion rate was adjusted to 20 ml/min with a peristaltic pump (Gilson Inc, USA). Coronary perfusion pressure (CPP) was measured by a pressure transducer fixed on a side-port of the Langendorff set-up. Left ventricular developed pressure (LVDP) was measured by a fluid-filled balloon inserted into the left ventricle and fixed to a second pressure transducer. The volume of the balloon was adjusted once at the beginning of the experiment to obtain an end diastolic pressure (EDP) of 5–10 mm Hg. Both pressure transducers were coupled to a PowerLab 4e recording system (PanLab SA, Barcelona, Spain) to measure CPP, LVDP and EDP. HR was derived from the left ventricle pressure signals (González et al., 2011). These experiments were carried out in 10–15 isolated hearts from each experimental group.

At the end of the cardiac function experiments, a portion of the left ventricle of each heart was separated, frozen at −80°C and stored for Western Blot determinations. Another portion of the left ventricle was processed for histological analysis.

### Aortic Ring Preparations

The aorta was carefully excised and placed in ice-cold Krebs-Henseleit (K-H) solution with the following composition (mM) (118 NaCl; 4.75 KCl; 1.2 MgSO_4_; 1.19 KH_2_PO_4_; 2.54 CaCl_2_; 25 NaHCO_3_; 11 glucose). All connective and perivascular adipose tissues were removed, with care taken not to disrupt the endothelium. Transverse vascular rings 3–4 mm long were prepared. The rings were fixed vertically between two stainless steel hooks and suspended in a 5-ml jacketed glass organ bath containing K-H buffer at 37°C and continuously bubbled with 95% O_2_ and 5% CO_2_. The upper wire was connected to an isometric force transducer (Grass FT07) and tension measurements were recorded on a computer (PowerLab/4e program). The rings were mounted with a resting tension of 2 g. Tissues were equilibrated for 90 min, during which time the medium was replaced every 15 min.

The aorta contractile and relaxant functions from animals of all the experimental groups were tested. To assess contractile function, phenylephrine (Phe) (10^−9^ M–10^−5^ M) concentration-response curves were performed. To evaluate vascular endothelium-dependent- and independent relaxation, carbachol (10^−9^ M–10^−4^ M) or sodium nitroprusside (SNP) (10^−9^ M–10^−6^ M) concentration-response curves, respectively, were established in Phe (1 μM) (submaximal) precontracted preparations (Abboud et al., 2009). Only one concentration-response curve was carried out in each preparation. The different experiments of vascular reactivity were performed in four rings of each aorta obtained from the animals of the different experimental groups.

Contraction responses of the aorta rings are expressed as mean absolute values and relaxation responses are expressed as the percentage relaxation of the tone induced by Phe.

A portion of aorta was separated, frozen at −80°C and stored for Western Blot determinations. Another portion of aorta was processed for histological analysis.

### Mesenteric Perfused Bed Preparation

The abdominal cavity was opened and the superior mesenteric artery was dissected and cannulated with a blunted hypodermic needle (21 G). The mesenteric bed was perfused with 50 ml of K-H solution containing 1000 IU of heparin and was separated from the gut by carefully cutting close to the intestinal wall. The preparation was then placed on a plate (8 cm × 8 cm) in a humid chamber and perfused with K-H at a constant flow rate of 5 ml/min, using a peristaltic pump (Gilson S.A.). The solution was maintained at 37°C and continually oxygenated (95% O_2_/5% CO_2_). Mesenteric vascular responses were detected as changes in perfusion pressure (mm Hg). This was monitored continuously using a pressure transducer (Transpac IV, Abbot) and recorded using a PowerLab (Powerlab 400, ADinstruments). The preparation was equilibrated for 30 min before experimentation.

The experiments were carried in intact mesenteric beds. The evaluation of functionality of the vascular bed was carried out following different procedures. Mesenteric bed contractile function (Carvalho Leone and Coelho, 2004) was evaluated by a concentration response curve of Phe (10–80 nmol). The vasorelaxant function was evaluated on precontracted bed with a concentration of Phe sufficient to increase the basal perfusion pressure by 60–100 mmHg. The endothelium-dependent vasodilatation was evaluated with a concentration-response curve of carbachol (3 × 10^−10^ mol–3 × 10^−5^ mol) and endothelium-independent vasodilatation was evaluated to a concentration-response curve of SNP (10^−11^mol–10^−6^mol). Doses of different drugs were injected in bolus with a Hamilton syringe (volume 50 μl) (Paniagua et al., 2016). These experiments were carried out in 10–15 isolated mesenteric beds from each experimental group.

Contraction responses of superior mesenteric artery bed are expressed as mean absolute values and relaxation responses are expressed as the percentage relaxation of the tone induced by Phe.

### Histological Analysis

At the end of the experimental period, samples of heart left ventricles and aorta rings were obtained from six animals per experimental group, fixed in buffered 10% formalin and embedded in paraffin. Histological damage and fibrosis were evaluated in sections 5 μm wide stained with hematoxylin-eosin stain (HE) and Masson’s trichrome stain for collagen fibers, or prepared for immunohistochemistry. Samples were studied under a Zeiss Axioskop 2 microscope equipped with the image analysis software package AxioVision 4.6. The analysis was made by triplicate in 5–8 random per section and specimen fields under a 20× or 40× objective. The experimenter was blind to the treatment received by the rat from which the sample under analysis was obtained.

For immunohistochemistry, samples were washed with phosphate buffered saline (PBS) with 0.05% Tween 20 (Calbiochem, Darmstadt, Germany). Thereafter sections were incubated for 10 min in 3% (vol vol^−1^) in hydrogen peroxide to inhibit endogenous peroxidase activity and blocked with 1% PBS-BSA (bovine serum albumin) or calf serum for 30 min to minimize non-specific binding of the primary antibody. Pilot experiments performed to determine the optimal antibody dilution showed that some samples needed to be pretreated by boiling in 10 mM citrate buffer for 30 min. Sections were then incubated overnight at 4°C with the following antibodies: monoclonal mouse anti-human connexin-43 (1:800; Santa Cruz Biotechnology, Santa Cruz, CA, USA); polyclonal rabbit anti-human eNOS (1:50; Novus Biologicals). After incubation, samples were washed with PBS-Tween. The peroxidase-based kit Masvision (Master Diagnostica, Granada, Spain) was used as secondary antibody. Samples were counterstained with hematoxylin and coverslips mounted with Eukitt mounting media (O. Kindler GmbH & Co., Freiburg, Germany). To determine the level of non-specific staining, the preparations were incubated without the primary antibody.

### Western Blot Analysis

For protein extraction, cardiac and aorta tissues (6–8 samples from 6 to 8 animals per group) were homogenized with ice-cold RIPA buffer containing 1 mM EGTA, 1 mM Na_3_VO_4_, 1 mM Na_4_P_2_O_7_, 10 mM NaF and a protease inhibitor cocktail (Roche, Spain). The homogenates were centrifuged (9300 × *g*, 10 min, 4°C) and the supernatant was extracted. Total protein values were quantified from all preparations using the Bradford method (Somoza et al., 2007).

Aorta (50 μg) and left ventricle (40 μg) samples were separated by electrophoresis on a 4–20% (aorta) or 10% (left ventricle tissue) Mini-protean^®^ TGX^TM^ Precast Gel (Bio-Rad, Spain) and transferred onto a PVDF membrane. The membranes were blocked with 3% fat-free milk at room temperature for 1 h and then incubated at 4°C overnight with primary antibody as follows: eNOS 1:250 (aorta), 1:500 (left ventricle tissue; BD Transduction Laboratories), connexin-43 (1:10000; left ventricle tissue; Santa Cruz Biotechnology, Santa Cruz, CA, USA), plasminogen activator inhibitor-1 (PAI-1) 1:500 (aorta, left ventricle tissue; Abcam, Cambridge, UK) and GAPDH 1:1000 (aorta, left ventricle tissue); Santa Cruz Biotechnology, Santa Cruz, CA, USA). These incubations were followed by incubation for 1 h at room temperature with an alkaline phosphatase-conjugated goat anti-mouse secondary antibody (1:10000) and subsequent treatment with ECF reagent (Thermo Fisher Scientific, USA). Protein bands were detected using Typhoon 9210 (GE Healthcare Life Sciences, USA) and the band intensity was assessed with ImageJ software (National Institutes of Health, USA). GAPDH was used as an internal control.

### Quantitative Real-time PCR Analysis

mRNAs were evaluated by quantitative real-time PCR. Total RNA was extracted from frozen cardiac and aorta tissues in Trizol reagent according to the manufacturer’s specifications (Invitrogen Life Technologies). The RNA concentrations were calculated by measuring the absorbance readings using Nanodrop Spectrophotometer ND-1000 (Nanodrop Technologies Inc., Willington, DE, USA). A total of 1 μg of RNA was reverse transcribed into cDNA using the High Capacity cDNA Archive Kit (Applied Biosystems) according to manufacturer’s instructions in a 20 μl reaction. PCR was performed in duplicate for each sample using 4.5 μl of diluted cDNA as template, 0.5 μl of 20x specific primers/probe mix and 2x SSO Advanced Universal Probes Supermix (Bio-Rad) in a 10 μl reaction. The amplification was carried out in an ABI 7500 Fast System (Life Technologies) by using the following conditions: 30 s, 95°C, 40 cycles (10 s 95°C, 30 s 60°C) in fast mode. As specific oligonucleotide primers and Taqman probes to detect amplification, we used Taqman Gene Expression Assays (Life Technologies): eNOS (Rn02132634_s1) and connexin-43 (Rn01433957_m1). As normalizing internal control, we amplified ribosomal 18S RNA. These determinations were carried out in five samples from five animals per group.

### Statistical Analysis

Data represent the mean ± SEM of observations obtained from 10 to 15 animals for *in vivo* and *in vitro* studies and 5–8 preparations (from different animals) for histological, Western Blot and quantitative real-time PCR analysis (*n*). Statistically significant differences were determined using by two-way or one-way analysis of variance followed by Bonferroni/Dunn *post hoc* test (Prism 4). *P*-values ≤ 0.05 were considered significant.

### Drugs

Cisplatin, phenylephrine, carbachol, and sodium nitroprusside were obtained from Sigma (Sigma Chemical Company, Poole, Dorset, UK).

Phenylephrine, carbachol, and sodium nitroprusside were dissolved in distilled water. Cisplatin was dissolved in saline (0.9% NaCl).

## Results

### Effect of Chronic Cisplatin Treatment on Blood Pressure and Heart Rate

**FIGURE 1:**
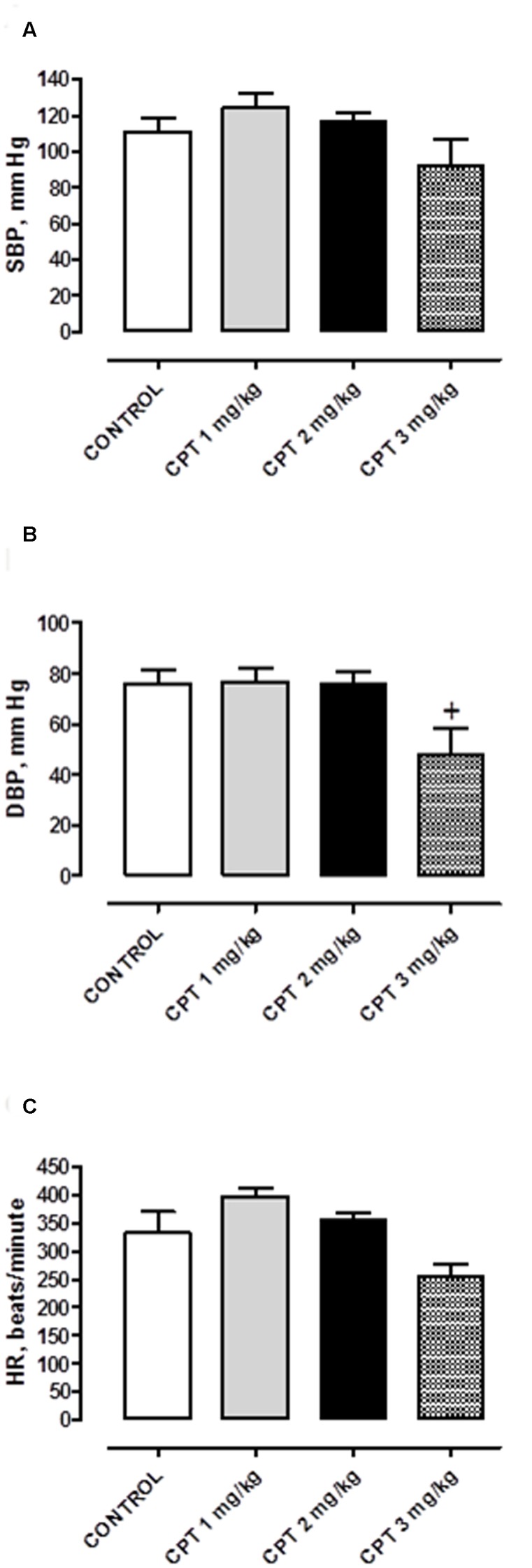
**(A)** Systolic blood pressure (SBP), **(B)** diastolic blood pressure (DBP), and **(C)** heart rate (HR) of anesthetized animals chronically treated with saline (CONTROL) or cisplatin (CPT) 1 mg/kg, 2 mg/kg or 3 mg/kg. Data represent the mean ± SEM, *n* = 10–15 animals per experimental group. A one-way ANOVA followed by Bonferroni/Dunn *post hoc* test was used for statistical analysis (^+^*P* < 0.05, CPT 3 mg/kg vs. control).

**Figure 1** shows the SBP, DBP, and HR in anesthetized rats after repeated treatment with saline or cisplatin at the three different doses evaluated.

Saline-treated rats had normotensive values for SBP (110.72 ± 8.10 mmHg, *n* = 15) and DBP (76.26 ± 5.35 mm Hg, *n* = 15) and normal HR (332.28 ± 37.82 beats/minute, *n* = 15). Cisplatin treatment caused only blood pressure alterations at the maximum dose administered (15 mg, cumulative dose), provoking, at this dose, a significant decrease in DBP (47.53 ± 11.08 mmHg, *n* = 10 *P* < 0.05 vs. control saline group) without modifying SBP (92.14 ± 14.15 mmHg, *n* = 10 *P* > 0.05 vs. control saline group) and HR (254.72 ± 20.82 beats/minute, *n* = 10 *P* > 0.05 vs. control saline group) (**Figure 1**).

### Effect of Chronic Cisplatin Treatment on Heart Function

**FIGURE 2:**
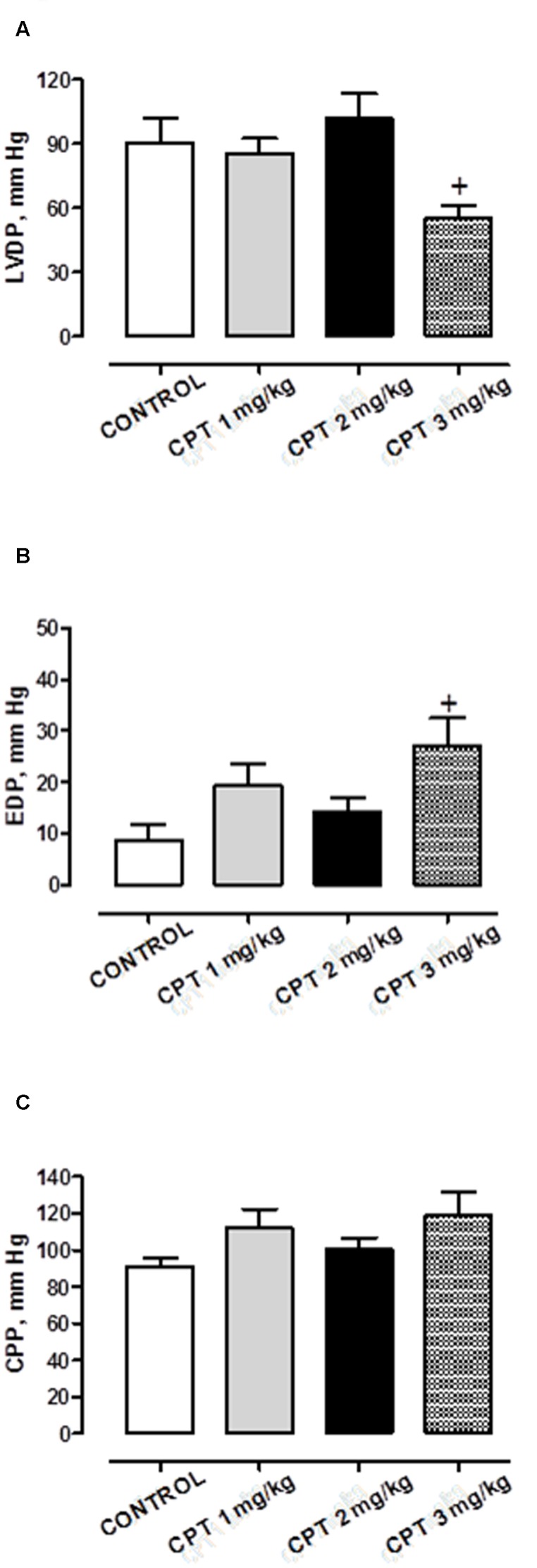
**Basal values of (A)** left ventricular developed pressure (LVDP), **(B)** end diastolic pressure (EDP), and **(C)** coronary perfusion pressure (CPP) of isolated hearts from animals chronically treated with saline (CONTROL) or cisplatin (CPT) 1 mg/kg, 2 mg/kg or 3 mg/kg. Data represent the mean ± SEM, *n* = 10–15 preparations per experimental group. A one-way ANOVA followed by Bonferroni/Dunn *post hoc* test was used for statistical analysis (^+^*P* < 0.05, CPT 3 mg/kg vs. control).

**Figure 2** shows the baseline cardiac values of left ventricle function (LVDP and EDP) and coronary flow (CPP) in the different groups of rats treated with saline or cisplatin at the three doses evaluated.

In saline treated rats, the LDVP was 90.57 ± 11.10 mmHg, *n* = 12, the EDP was 8.79 ± 2.98 mmHg, *n* = 12 and the CPP was 90.91 ± 5.21 mmHg, *n* = 12. Cisplatin only caused significant cardiac alterations at the maximum dose administered (15 mg, cumulative dose), provoking at this dose, a significant decrease in contractile and relaxant function (as the increase in EDP showed) of the left ventricle (LVDP and EDP, respectively) (LVDP: 55.45 ± 6.09 mmHg, *n* = 10 *P* < 0.05; EDP: 27.03 ± 5.51 mmHg, *n* = 10 *P* < 0.05 vs. control saline group) without affecting CPP in comparison with saline treated rats (CPP: 119.17 ± 12.19 mm Hg, *n* = 10 *P* > 0.05 vs. control saline group).

### Effect of Chronic Cisplatin Treatment on Aortic Vascular Function

**FIGURE 3:**
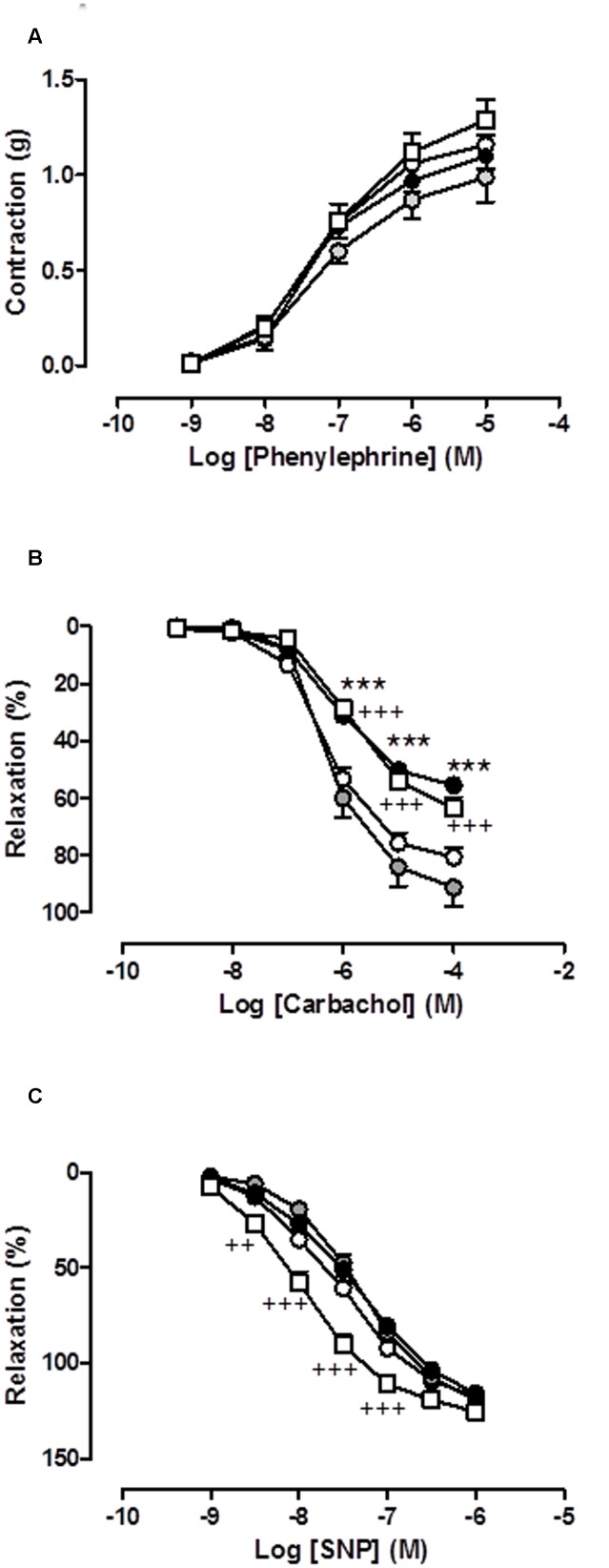
**(A)** Concentration-response curve of phenylephrine (10^−9^ M–10^−5^ M), **(B)** concentration-response curve of carbachol (10^−9^ M–10^−4^ M), and **(C)** concentration-response curve of sodium nitroprusside (SNP) (10^−9^ M–10^−6^ M) in isolated rat aorta rings from animals chronically treated with saline (CONTROL, white circles) or cisplatin (CPT) 1 mg/kg (gray circles), 2 mg/kg (black circles) or 3 mg/kg (white squares). Values are expressed as mean ± SEM, *n* = 10–15 preparations per experimental group. A two-way ANOVA followed by Bonferroni/Dunn *post hoc* test was used for statistical analysis (^∗∗∗^*P* < 0.001, CPT 2 mg/kg vs. control; ^++^*P* < 0.01, ^+++^*P* < 0.001 CPT 3 mg/kg vs. control).

**Figure 3** shows the contractile function (measured as response to Phe), the endothelial-dependent relaxation (measured as response to carbachol) and the endothelial-independent relaxation (measured as response to SNP) in the isolated aorta (a conduit vessel) of the rats from the different groups treated with saline or cisplatin at the three doses evaluated.

In the saline-treated group, Phe provoked a concentration-dependent increase in aortic vascular tone resulting in an *R*_max_ value of 1.16 ± 0.05 g (*n* = 15), carbachol caused an endothelial-dependent concentration-dependent decrease in aortic vascular tone resulting in a *R*_max_ value of 80.70 ± 3.30% (*n* = 15), and SNP caused an endothelial-independent concentration-dependent decrease in aortic vascular tone resulting in a *R*_max_ value of 117.74 ± 2.01% (*n* = 15). The weekly chronic administration of cisplatin at the three doses assayed, 1, 2, and 3 mg/kg for 5 weeks (cumulative dose of 5, 10, and 15 mg, respectively) did not affect the aortic vasoconstrictor response to Phe (**Figure 3A**), but provoked a clear and significant inhibition of the carbachol-mediated vasorelaxant response at the doses of 2 and 3 mg/kg, but not at the dose of 1 mg/kg of cisplatin. It is important to mention that the inhibition of the endothelial dependent vasorelaxation was similar at the two higher cisplatin doses evaluated (2 and 3 mg/kg) (**Figure 3B**). However, the endothelial-independent vasorelaxation was only affected by chronic treatment with cisplatin at the maximum dose assayed (3 mg/kg), which showed a significant potentiation of this vasorelaxation in aorta rings from rats treated with this particular dose of the antitumoral agent (**Figure 3C**).

### Effect of Chronic Cisplatin Treatment on Mesenteric Vascular Function

**FIGURE 4:**
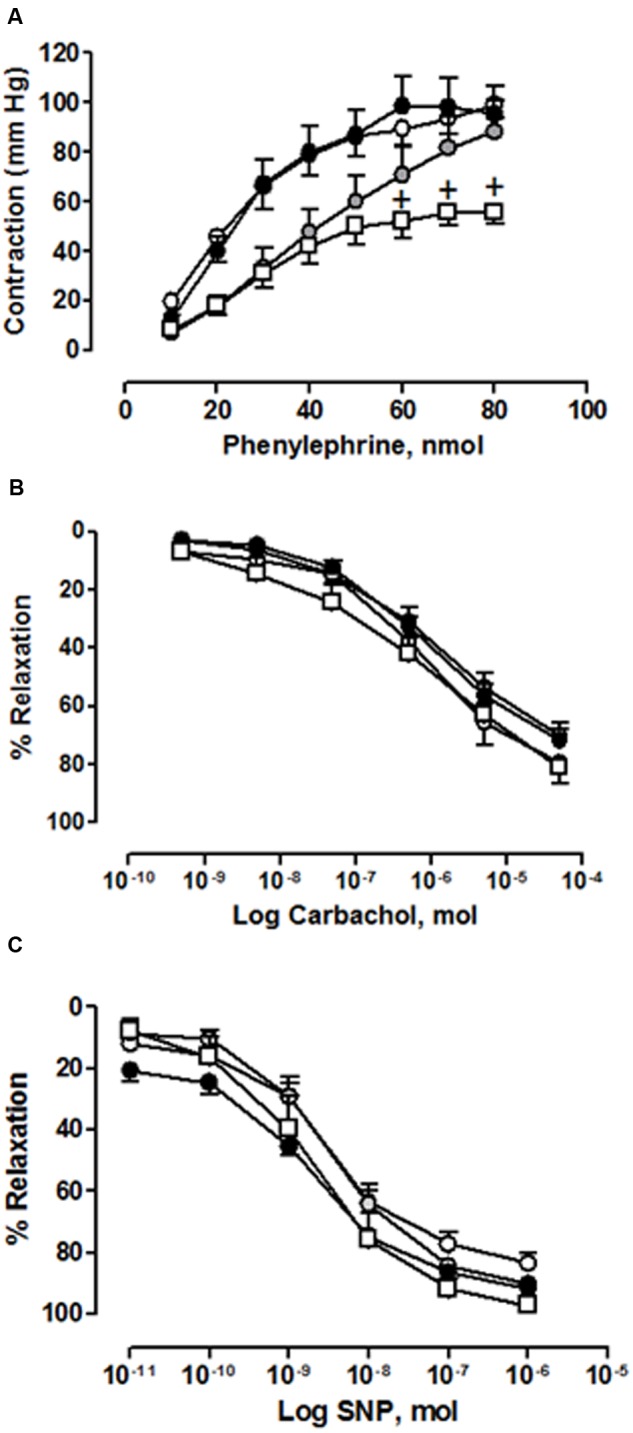
**(A)** Concentration-response curve of phenylephrine (10 nmol–80 nmol), **(B)** concentration-response curve of carbachol (3 × 10^−10^mol–3 × 10^−5^ mol) and **(C)** concentration-response curve of sodium nitroprusside (SNP) (3 × 10^−11^mol–3 × 10^−6^ mol) in perfused mesenteric bed from animals chronically treated with saline (CONTROL, white circles) or cisplatin (CPT) at 1 mg/kg (gray circles), 2 mg/kg (black circles) or 3 mg/kg (white squares). Values are expressed as mean ± SEM, *n* = 10–15 preparations per experimental group. A two-way ANOVA followed by Bonferroni/Dunn *post hoc* test was used for statistical analysis (^+^*P* < 0.05, CPT 3 mg/kg vs. control).

**Figure 4** shows the contractile function (measured as response to Phe), the endothelial-dependent relaxation (measured as response to carbachol) and the endothelial-independent relaxation (measured as response to SNP) in the perfused mesenteric bed (a resistance vascular territory) of the rats from the different groups treated with saline or cisplatin at the three doses evaluated.

In the saline-treated group, Phe provoked a concentration-dependent increase in mesenteric vascular tone resulting in an *R*_max_ value of 99.13 ± 5.45 mm Hg (*n* = 10), carbachol caused an endothelial-dependent concentration-dependent decrease in mesenteric vascular tone resulting in a *R*_max_ value of 70.70 ± 4.07% (*n* = 12), and SNP caused an endothelial-independent concentration-dependent decrease in mesenteric vascular tone resulting in a *R*_max_ value of 83.47 ± 3.02% (*n* = 10). The results obtained in this representative resistance territory were different from those obtained in the conduit vessel, the aorta. The weekly chronic administration of cisplatin provoked a significant decrease in the mesenteric vasoconstrictor response to Phe at the highest dose of cisplatin assayed (3 mg/kg) (**Figure 4A**), but did not provoke any modification in the endothelial-dependent or independent vasorelaxant function in mesenteric bed at any of the cisplatin doses evaluated (**Figures 4B,C**).

### Cardiovascular Alterations Induced by Chronic Cisplatin Treatment: Structural and Molecular Mechanisms Involved

**FIGURE 5:**
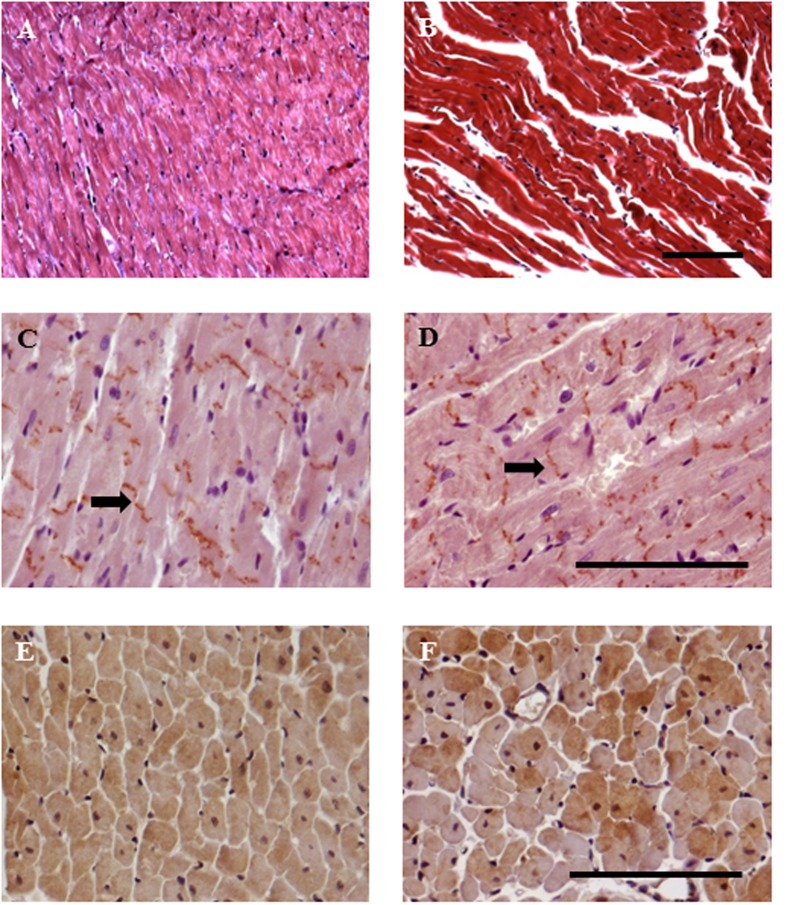
**Representative images of histology and immunohistochemistry of the effect of cisplatin treatment on rat hearts.** Rats were injected intraperitoneally for 5 weeks with saline (0.9% NaCl, left column) or cisplatin (3 mg/kg week^−1^, right column). Histological samples embedded in paraffin and stained with Masson’s trichrome **(A,B)**. Samples processed for immunohistochemistry with anti connexin-43 antibody (black arrows) **(C,D)** and with anti eNOS **(E,F)**. Heterogenicity in fiber staining is clearly seen. Bar: 100 μm.

**FIGURE 6:**
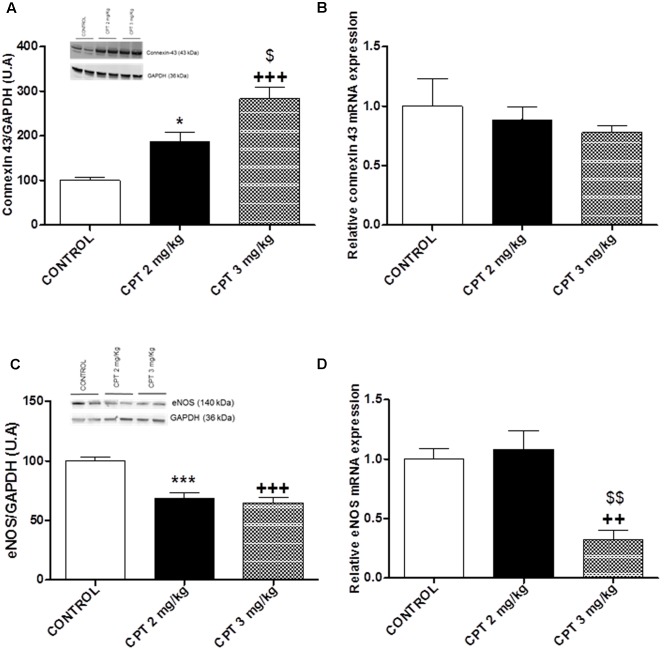
**(A)** Representative immunoblots for connexin-43 protein expression in whole cardiac left ventricle. Diagram bars show the results of densitometric analysis of connexin-43 in whole cardiac left ventricle. Homogenized samples from heart show essentially the connexin-43 in its phosphorylated form (strongest bands for the quantification) **(B)** Quantitative analysis of connexin-43 mRNA levels in whole cardiac left ventricle. **(C)** Representative immunoblots for eNOS protein expression. Diagram bars show the results of densitometric analysis of eNOS in whole cardiac left ventricle. **(D)** Quantitative analysis of eNOS mRNA levels in whole cardiac left ventricle Data are presented as means ± SEM of observations obtained for 5–8 tissue samples from 5 to 8 animals per treatment. A one-way ANOVA followed by Bonferroni/Dunn *post hoc* test was used for statistical analysis (^∗^*P* < 0.05, CPT 2 mg/kg vs. control, ^∗∗∗^*P* < 0.001, CPT 2 mg/kg vs. control, ^+++^*P* < 0.001, CPT 3 mg/kg vs. control, ^$^*P* < 0.05, CPT 3 mg/kg vs. CPT 2 mg/Kg, ^$$^*P* < 0.01, CPT 3 mg/kg vs. CPT 2 mg/Kg).

Cisplatin treatment evoked a clear damage in the structure of cardiac fibers (**Figures 5A,B**). Normal cardiac architecture was altered when increasing the dose of cisplatin, appearing rippled cardiomyocytes and fibrils de-arrangement. Similarly, connexin-43 expression was also modified showing a more intense and more diffused location with the higher dose of cisplatin, 3 mg/kg (**Figures 5C,D**). Western blot analysis also revealed that the expression of connexin-43 in cardiac left ventricle was significantly increased at the cisplatin doses of 2 mg/kg (*P* < 0.05) and 3 mg/kg compared with the control group (*P* < 0.001) (**Figure 6A**). Furthermore, connexin-43 expression was significantly increased in the cisplatin 3 mg/kg group in comparison with the cisplatin 2 mg/kg group (*P* < 0.05). However, no changes in connexin-43 mRNA levels were detected at the two different doses of cisplatin evaluated in comparison with control group (**Figure 6B**). On the other hand, histological preparations showed that cardiac eNOS expression was more heterogeneous after cisplatin treatment (**Figures 5E,F**). Western blot analysis showed that this eNOS expression was significantly decreased at the doses of cisplatin 2 mg/kg and 3 mg/kg compared with the control group (*P* < 0.001) (**Figure 6C**), while eNOS mRNA level was significantly decrease only at the dose of 3 mg/kg, but not at the dose of 2 mg/kg compared with the control group (*P* < 0.01) (**Figure 6D**).

**FIGURE 7:**
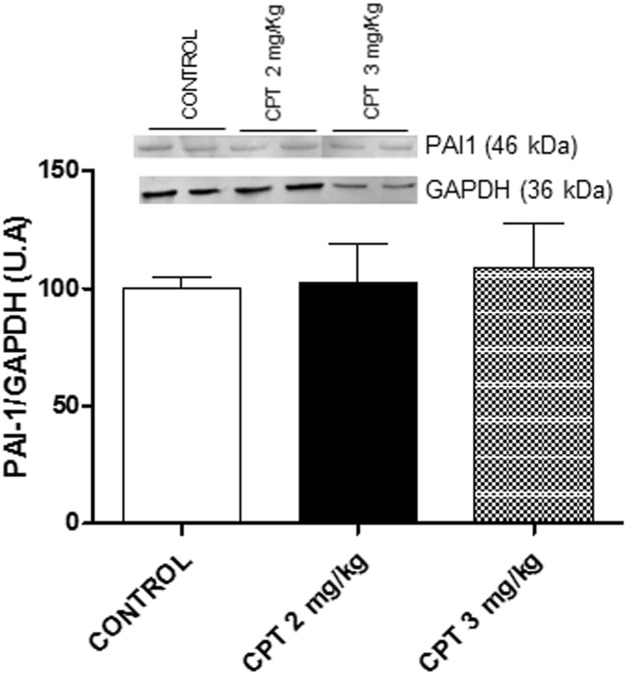
**Representative immunoblots for plasminogen activator inhibitor-1 (PAI-1) protein expression in whole cardiac left ventricle.** Diagram bars show the result of densitometric analysis of PAI-1 in whole cardiac left ventricle. Data are presented as means ± SEM of observations obtained of 6–8 tissue samples from 6 to 8 animals per treatment. A one-way ANOVA followed by Bonferroni/Dunn *post hoc* test was used for statistical analysis.

An elevated level of PAI-1 is also an important diagnostic marker of cardiac fibrosis (Ghosh and Vaughan, 2012). For that, the cardiac expression of PAI-1 was analyzed in this study. There was not any change in the cardiac expression of PAI-1 in both, cisplatin 2 and 3 mg/kg treated animals with respect to the control group (*P* > 0.05) (**Figure 7**).

**FIGURE 8:**
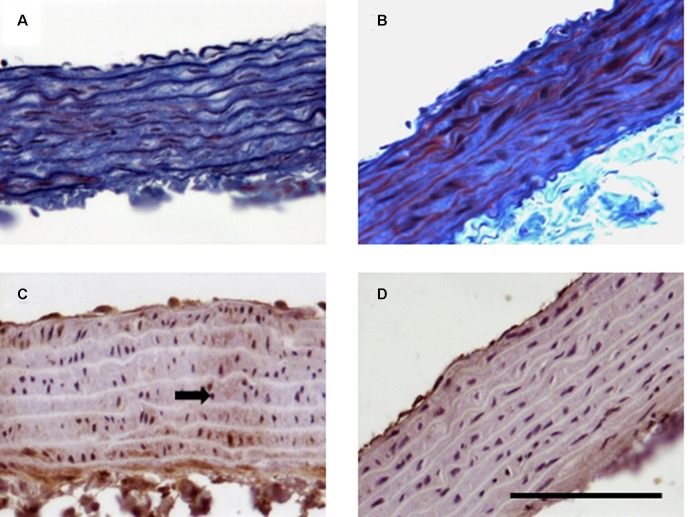
**Representative images of histology and immunohistochemistry of the effect of cisplatin treatment on rat aorta.** Rats were injected intraperitoneally for 5 weeks with saline (0.9% NaCl, left column) or cisplatin (3 mg/kg week^−1^, right column). Histological samples embedded in paraffin and stained with Masson’s trichrome **(A,B)**. Samples processed for immunohistochemistry with anti eNOS antibody **(C,D)**. Bar: 100 μm.

**FIGURE 9:**
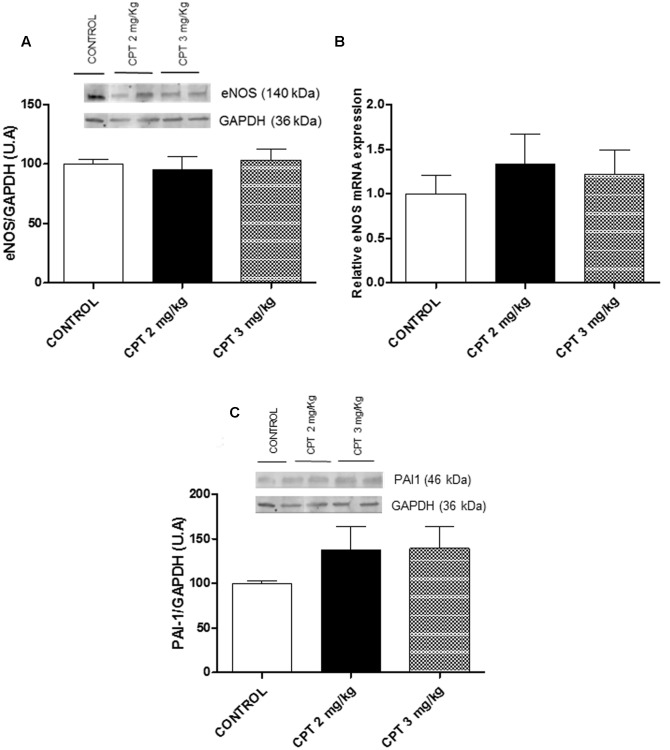
**(A)** Representative immunoblots for eNOS protein expression in aorta. Diagram bars show the result of densitometric analysis of eNOS in aorta. **(B)** Quantitative analysis of eNOS mRNA levels in aorta. **(C)** Representative immunoblots for plasminogen activator inhibitor-1 (PAI-1) protein expression in aorta. Diagram bars show the result of densitometric analysis of PAI-1 in aorta. Data are presented as means ± SEM of observations obtained for 5–8 tissue samples from 5 to 8 animals per treatment. A one-way ANOVA followed by Bonferroni/Dunn *post hoc* test was used for statistical analysis.

Regarding the aorta, the morphological analysis showed changes in the fiber arrangement in the tunica media, losing their uneven appearance (**Figures 8A,B**). eNOS staining was strongly located in endothelium with certain positive areas in tunica media that disappeared in the animals treated with cisplatin 3 mg/kg (**Figures 8C,D**). However, the cisplatin treatments did not provoke any modification either in aorta expression of eNOS or in the eNOS mRNA levels in comparison with the control group (*P* > 0.05) (**Figures 9A,B**). The expression on PAI-1 was also analyzed in aorta tissue in the different experimental groups. Aortic expression of PAI-1 was slightly, but not significantly, increased at the two different doses of cisplatin evaluated in relation to the control group (**Figure 9C**).

## Discussion

This study shows that the chronic treatment with cisplatin causes dose-dependent cardiovascular alterations. Vascular toxicity occurs at lower doses than cardiac or systemic cardiovascular alterations. The cisplatin induced-vascular toxicity could be cataloged as “silent” because it occurs even in the absence of any systemic cardiovascular modifications. At high doses of cisplatin treatment, vascular toxicity is maintained and cardiac and blood pressure alterations are present. Cisplatin treatment also produced alterations in the structure of cardiac and vascular tissue, suggesting a direct cytotoxicity. Changes in connexin-43 and eNOS expression could be related with cardiac functional alterations after cisplatin treatments.

In recent years, many clinical studies have noted that some cisplatin-treated cancer survivors have a significantly increased risk of cardiovascular events (Ishioka et al., 2008; Vaughn et al., 2008), cisplatin-induced cardiovascular toxicity being an increasing concern. Although, there are some experimental data in cardiac toxicity induced after acute administration of cisplatin (Al-Majed et al., 2006; Yüce et al., 2007), there are no experimental studies specifically evaluating cardiovascular alterations after chronic cisplatin treatment, and studying, at the same time, mechanisms involved in these particular alterations.

As a first approximation, the present study evaluated general cardiovascular parameters (blood pressure and HR) in different cisplatin chronic treatments (1, 2, and 3 mg/kg). The results show that, at the highest dose of the antitumor drug used, a significant decrease in the DBP and a slight, although not significant bradycardia occur. SBP was not modified after treatments, and HR was not altered after low and intermediate cisplatin doses assayed. It was demonstrated that cisplatin chronic treatment at similar doses (1–3 mg/kg) than those evaluated in the present work caused peripheral sensory and enteric autonomic neuropathy (Vera et al., 2011, 2013). Our results show that cisplatin chronic treatment could also provoke an autonomic cardiovascular neuropathy resulting in a decrease in DBP and slight bradycardia, pointing out that this cardiovascular autonomic neuropathy occurs at higher doses than the sensory or enteric neuropathies that have been shown at treatment doses of cisplatin of 1 and 2 mg/kg (Vera et al., 2011, 2013). Different authors describe that cisplatin induced severe bradycardia in humans (Darling, 2015; Schlumbrecht and Hehr, 2015; Kounis et al., 2016), and other authors describe, after a single injection of cisplatin (7 mg/kg) in rats, cardiac alterations that include a decrease in blood pressure as well as a decrease in HR, due to the cardiotoxicity that cisplatin produces in the sinoatrial node, which leads to bradycardia (El-Sawalhi and Ahmed, 2014). On the other hand, cisplatin causes nephrotoxicity primarily causing tubulointerstitial lesions that provoke the urine waste of electrolytes (Vickers et al., 2004; Yao et al., 2007). Besides, it has also been demonstrated that most cisplatin-treated patients waste sodium, potassium, magnesium, and calcium in their urine and some have orthostatic hypotension (Lajer and Daugaard, 1999; Goren, 2003). In our study, renal toxicity has not been evaluated, but it is possible that both phenomena, cardiac and renal toxicities, were also related. In fact, understanding the mechanisms of cisplatin-induced renal and cardiac toxicities may help provide better treatment and preventive strategies (Dugbartey et al., 2016). More research is needed to investigate this aspect. It is important to note that the fact that neither SBP nor HR were modified could suggest that we detected cisplatin-induced cardiovascular neurotoxicity still at an initial developmental stage.

In cancer patients treated with chemotherapy, there is greater cardiovascular morbidity and mortality than in the general population, especially and worryingly in patients younger than 45 (van den Belt-Dusebout et al., 2006). The most evaluated cardiac toxicity is that produced by anthracyclines (Hirano et al., 1993; Soga et al., 2006; Berdichevski et al., 2010; Shaker and Sourour, 2010). However, this cardiotoxicity is not unique to this group of antineoplastic drugs, but may occur with other antitumoral agent groups (Al-Majed et al., 2006; Drimal et al., 2006; Hernández-Esquivel et al., 2006; Sudharsan et al., 2006), including cisplatin (Pai and Nahata, 2000). It was reported that treatment with a single dose of cisplatin causes left ventricular dysfunction and depression of cardiomyocyte contractility in the rat. These alterations may be related to mitochondrial function, oxidative stress and an increase in apoptosis (Ma et al., 2010; El-Sawalhi and Ahmed, 2014). The results obtained in this study confirm that chronic cisplatin produces a significant decrease in cardiac contractility, associated with a decrease in dilatory left ventricular function. Moreover, this left ventricular dysfunction could be related to the decrease in DBP observed after cisplatin at 3 mg/kg/week. Other authors, using similar or even lower doses but in an acute administration pattern, obtained similar results (Ma et al., 2010; Ciftci et al., 2011; El-Awady et al., 2011; Hussein et al., 2012; Coskun et al., 2014; El-Sawalhi and Ahmed, 2014).

In this study, it has been also evaluated the possible mechanism involved in the left ventricular dysfunction observed after cisplatin treatment. Connexins are structural proteins that bind to form gap bonds in vertebrates, and connexin-43 is a member of this family. Gap junctions allow the passage of small ions and molecules, directly connecting the cytoplasm of adjacent cells, and are of special importance in the heart, since they make possible the coordinated depolarization of the cardiac muscle. An increase in the expression of connexin-43 has been described in cisplatin-resistant tumor cell lines, which seems to be linked to the resistance of the cells to this drug (Li et al., 2006). However, the expression of this protein in cardiac tissue of animals treated with cisplatin has not been extensively studied. Our results show that cardiac architecture was altered when increasing the dose of cisplatin, with occurrence of rippled cardiomyocytes and fibrils de-arrangement. Moreover, an increase in connexin-43 expression in hearts of cisplatin-treated animals was observed with the dose of 2 mg/kg and higher. This data suggests that cisplatin can produce direct cytotoxicity in cardiac cells and that they can respond with an increase of connexin-43. Other authors have also described the cytotoxicity of acute (10 mg/kg) or chronic (4 mg/kg one a week for 4 weeks) administration of cisplatin that resulted in structural alterations in cardiac tissue in which separated cardiac muscles with interruption of myofibrils and severe interstitial hemorrhages or fibrosis were observed (Jiang et al., 2014; Saleh et al., 2015). It is important to note that connexin-43 RNAm levels were not modified after the antitumoral treatments. It is possible that alterations in some transcription and post-transcriptional factors or inhibition of connexin-43 degradation play a role in the regulation of connexin expression (Salameh et al., 2004).

It is known that an elevated level of PAI-1 is also an important profibrotic marker of cardiac fibrosis (Ghosh and Vaughan, 2012); and that an increased expression of myocardium PAI-1 contributes to ventricular remodeling and fibrosis that could be related to ventricular dysfunction (Takeshita et al., 2004; Shimizu et al., 2016). The result of the present study show that PAI-1 cardiac expression was not modified after the different cisplatin treatments, what could suggest that cardiac alterations in our experimental protocol were at an incipient stage. Other authors have described an increase of PAI-1 in cardiomyocytes treated with other chemotherapeutic agents as doxorubicin (Ghosh et al., 2016).

On the other hand, heart eNOS was heterogeneously distributed and eNOS expression and eNOS mRNA levels were diminished after cisplatin treatment at the higher doses assayed. Our data are in agreement with those showed by Saleh et al. (2015) that also described a significantly decrease in nitric oxide levels in heart homogenates after chronic cisplatin treatments. However, after acute cisplatin administration, other authors show a significant increase in the level of nitric oxide in heart (Jiang et al., 2014). It is possible that the repeated pattern of administration used in this study can ameliorate this increase. Besides, it is known that overproduction of nitric oxide was directly linked to heart damage in other models of chemotherapeutic agents-induced cardiotoxicity, as in cyclophosphamide (Mythili et al., 2004), and doxorubicin (Ghibu et al., 2012). However, our data suggest that chronic cisplatin treatment does not cause this increase. More research is needed to evaluate more deeply this alteration.

Regarding functionality of blood vessels, the results obtained showed that chronic cisplatin treatment provoked an altered vascular relaxant but not vascular contractile function in aorta, indicating the presence of a clear endothelial dysfunction in a large artery. This endothelial dysfunction did not occur at the low dose evaluated of the chemotherapeutic agent but was present at 2 and 3 mg/kg. It is important to note that the magnitude of this endothelial dysfunction is not dose-dependent since the doses of 2 and 3 mg/kg induced a similar decrease in the endothelial function. Endothelial dysfunction in large arteries has been also described in humans (Turlapaty and Altura, 1980; Rosenfeld and Broder, 1984; Samuels et al., 1987; Serrano-Castro et al., 2000; Pretnar-Oblak et al., 2007). On the other hand, endothelium-independent vasorelaxation in aorta was augmented by the maximum dose of cisplatin evaluated, which indicates a greater facility of cisplatin to produce direct relaxation in vascular smooth muscle at this dose. Recently, it has been described, in rats, that cisplatin (200 μM) decreased contractile function in thoracic aorta and that this effect was caused by a severe damage to blood vessel walls (Jiang et al., 2014). This fact could explain the potentiation of endothelial-independent vasorelaxation observed in our study in aorta after the administration of 3 mg/kg of cisplatin. However, this fact is in contrast with the endothelial dysfunction observed. The resistance vascular territory was differently affected by chronic cisplatin treatment. Thus, in the mesenteric vascular bed, cisplatin treatment did not provoke any modification in the vasorelaxant function at any of the three different cisplatin doses evaluated, but caused a significant reduction in the vasocontractile function of this vascular bed that is compatible with the existence of an autonomic neuropathy at this level. In fact, Authier and Coworkers pointed out that peripheral nerve conduction velocities were decreased in cisplatin (3 mg/kg) treated rats (Authier et al., 2003). Moreover, in cisplatin-treated patients a similar decrease in nerve conduction velocity occurred after cumulative doses of 200–400 mg/m^2^ (Boogerd et al., 1990). It is possible that cumulative cisplatin doses of 10 or 15 mg/kg are not high enough to produce this altered alpha-vasoconstrictor response in large vessels, whereas resistance territories may be more sensitive to its neuronal toxicity. In the literature, there is no experimental data about cisplatin toxicity on resistance vascular vessels, to which we could compare the result obtained in the present work. More research is needed to completely establish the differences found between resistance and large vascular territories in contractile function after cisplatin treatments.

Regarding the endothelial dysfunction observed only in large but not in resistance vessels, it is possible that vascular endothelial dysfunction in large vessels occurs prior to vascular autonomic neuropathy in cisplatin treatments. In addition, endothelial dysfunction may be more sensitive and therefore dysfunction may occur even at low doses. This pattern of events is not surprising. In fact, other authors have shown in other autonomic peripheral neuropathies, such as diabetic neuropathy, that impairment of acetylcholine-mediated vascular relaxation occurs prior to nerve blood flow and conduction deficits (Oltman et al., 2005).

Finally, in order to identify the possible molecular mechanism by which cisplatin caused the vascular toxicity described here, histological, Western Blot and quantitative PCR analyses were performed. Methodological problems have impeded us to carry out these experiments in mesenteric vessels, but they have been carried out in aorta. Structural alterations occurred in aorta after chronic cisplatin treatment. The results obtained show changes in fiber arrangement in the tunica media, losing their uneven appearance. The fundamental structural and functional unit of the aortic wall is the medial lamellar unit. The medial layer comprises elastic membrane layers between which the smooth muscle layer and a small amount of collagen and elastic fibers are encountered. The integrity of the vascular smooth muscle layer is crucial in the maintenance of normal vascular morphology and tone. Changes in arterial wall composition and function underlie all forms of vascular disease (Dabagh et al., 2008). Other authors have also described, after acute administration of cisplatin at 5 mg/kg, severe damage to the smooth muscle layer of aorta (Jiang et al., 2014). Furthermore, Saleh et al. (2015) also described an increased thickening in tunicae intima and media with an irregular luminal layer of endothelial cell linings in aorta from chronic cisplatin treated rats. In the present study, histological analysis also showed that eNOS staining disappeared in the animals treated chronically with cisplatin at 3 mg/kg. However, it was surprising that eNOs expression or eNOs RNAm levels were not altered in aorta after the different cisplatin treatments. It is possible that endothelial dysfunction also was in an incipient stage or that other mechanisms were involved in cytotoxic effect of cisplatin in blood vessels. It is known that there is a relationship between endothelial dysfunction and vascular PAI-1 upregulation (Endemann and Schiffrin, 2004; Vaughan, 2005). So, in the present work, the expression of PAI-1 was also evaluated in aorta. Cisplatin treatments induced slightly but not significant increases in PAI-1 expression in aorta tissue. More research is needed to determine the possible role of other mechanisms involved in cytotoxic effect of cisplatin in blood vessels.

## Conclusion

In summary, the results of this study show that chronic treatment with cisplatin induces cardiovascular alterations. These cardiovascular alterations do not affect equally all cardiovascular organs/tissues and they do not occur at the same doses of the antitumor treatment. Thus, at low doses, chronic treatment with cisplatin seems safe for the cardiovascular system, since no alterations are observed. However, at intermediate doses, alterations, such as endothelial dysfunction in vessels of conductance, are apparent. At higher doses, this endothelial dysfunction is maintained, and other alterations also develop. In this sense, the autonomic neurotoxicity begins affecting resistance vessels and cardiac function, and this finally causes general symptoms such as hypotension. These functional alterations at the cardiovascular level are accompanied by structural alterations in cardiac and vascular tissue. Changes in connexin-43 and eNOS expression could be related with cardiac functional alterations after cisplatin treatments.

Both traditional and novel anticancer agents are used with curative purpose. However, limiting cardiovascular problems of these therapeutic drugs has become a priority. In this sense, experimental studies directed to the identification and understanding of the mechanisms involved in this particular toxicity could be useful to the scientific community. In the present study, the experimental conditions used provoked in the animals, cardiovascular alterations similar to those described in humans treated with cycles of cisplatin (Demkow and Stelmaszczyk-Emmel, 2013; Haugnes et al., 2015; Herrmann et al., 2016), being a useful tool for this purpose. However, further investigations are needed to identify early signs of cardiac and vascular damage in order to optimize the clinical management of cardiotoxicity in cisplatin treatments.

## Author Contributions

VL-M designed the study. VL-M, EH, CG, JU, and RA performed the experiments and analyzed the data. VL-M, EH, and JU wrote the manuscript. MM contributed financial support and supervised research. All authors reviewed and approved the final version of the manuscript.

## Conflict of Interest Statement

The authors declare that the research was conducted in the absence of any commercial or financial relationships that could be construed as a potential conflict of interest.
